# Tumor Vascular Morphology Undergoes Dramatic Changes during Outgrowth of B16 Melanoma While Proangiogenic Gene Expression Remains Unchanged

**DOI:** 10.5402/2011/409308

**Published:** 2011-12-04

**Authors:** Elise Langenkamp, Franziska M. vom Hagen, Peter J. Zwiers, Henk E. Moorlag, Jan P. Schouten, Hans-Peter Hammes, Annette S. H. Gouw, Grietje Molema

**Affiliations:** ^1^Medical Biology Section, Department of Pathology and Medical Biology, University Medical Center Groningen, University of Groningen, 9713 GZ Groningen, The Netherlands; ^2^Department of Immunology, Genetics and Pathology, Rudbeck Laboratory, Uppsala University, 75185 Uppsala, Sweden; ^3^Fifth Medical Department, Medical Faculty Mannheim, Heidelberg University, 68167 Mannheim, Germany; ^4^Department of Epidemiology, University Medical Center Groningen, University of Groningen, 9713 GZ Groningen, The Netherlands; ^5^Department of Pathology, University Medical Center Groningen, University of Groningen, 9713 GZ Groningen, The Netherlands

## Abstract

In established tumors, angiogenic endothelial cells (ECs) coexist next to “quiescent” EC in matured vessels. We hypothesized that angio-gene expression of B16.F10 melanoma would differ depending on the growth stage. Unraveling the spatiotemporal nature thereof is essential for drug regimen design aimed to affect multiple neovascularization stages. We determined the angiogenic phenotype—represented by 52 angio-genes—and vascular morphology of small, intermediate, and large s.c. growing mouse B16.F10 tumors and demonstrated that expression of these genes did not differ between the different growth stages. Yet vascular morphology changed dramatically from small vessels without lumen in small to larger vessels with increased lumen size in intermediate/large tumors. Separate analysis of these vascular morphologies revealed a significant difference in **α**SMA expression in relation to vessel morphology, while no relation with VEGF, HIF-1**α**, nor Dll4 expression levels was observed. We conclude that the tumor vasculature remains actively engaged in angiogenesis during B16.F10 melanoma outgrowth and that the major change in tumor vascular morphology does not follow molecular concepts generated in other angiogenesis models.

## 1. Introduction

Tumors depend on the recruitment of new blood vessels to grow beyond a certain size and to metastasize to other tissues. This process represents an attractive target for the treatment of cancer, and several strategies have been pursued to inhibit neovascularization [1]. Yet, antiangiogenic drugs in clinical trials have not fully lived up to their expectations [2, 3]. The limited efficacy of antiangiogenic therapies may find its origin in heterogeneity of the molecular activation status of endothelial cells in the tumor vasculature [4, 5]. More than ten years ago, Bergers et al. demonstrated that the efficacy of antiangiogenic drugs in a multistage carcinogenesis model depends on the tumor growth stage being targeted [6]. Additional evidence for differential susceptibility of tumor vasculature to antiangiogenic treatment was given by Wood et al. [7]. They showed that inhibition of vascular endothelial growth factor- (VEGF-) receptor 2 caused a reduced occurrence of microvessels in the interior of the tumor, while larger, more mature vessels remained unaffected [7].

We hypothesize that the tumor growth stage-specific responses to antiangiogenic drugs are related to the existence of differences in angiogenic status of the tumor microvasculature in the different stages of tumor growth. As tumor endothelial behavior results from the local presence of growth factors, cytokines, and other molecules, which varies during tumor development [8–10], the differences in angiogenic status would be reflected in variations in local expression patterns of angiogenesis- and vascular maturation-related genes during tumor outgrowth. The basis for rational antiangiogenic therapy regimen design throughout the different stages of tumor growth lies in the understanding of the nature and location of the expression of angiogenic genes and local balances thereof in their pathophysiological environment.

While a vast number of studies investigated the molecular control of angiogenesis using transgenic animal models that either overexpress specific genes or have a full gene knock-out, only limited studies describe the molecular angiogenic make up of tumors that is endorsed by endogenously controlled mechanisms. In the current study we investigated the angiogenic phenotype of the widely used, subcutaneously (s.c.) growing mouse B16.F10 melanoma model at an early, intermediate and late stage of tumor growth. By real-time RT-PCR, we quantified the expression of 52 genes that have previously been reported to be major controllers of tumor angiogenesis. Based on the morphological differences observed, we next zoomed in on the tumor vascular compartment by laser microdissection, to obtain a more detailed view on the localization of angio-gene expression in the complexity of the *in vivo* tumor microenvironment. Lastly, we analyzed a number of vascular features, *that is*, vascular morphology, pattern of pericyte association, and proliferation status of tumor and endothelial cells in the different growth stages, and related them to the observed local gene expression patterns.

## 2. Materials and Methods

### 2.1. Tumor Cell Culture and Animal Studies

B16.F10 murine melanoma cells were cultured in 75 cm^2^ culture flasks in DMEM (Biowhittaker, Verviers, Belgium) supplemented with 10% fetal calf serum (FCS; Hyclone, Perbio Science, Etten-Leur, The Netherlands), 2 mM L-glutamine (Biowhittaker), and 1% gentamycin (Biowhittaker) at 37°C and 5% CO_2_/95% air in a humidified incubator.

Male C57bl/6 mice (20–25 g; Harlan, Zeist, The Netherlands) were subcutaneously inoculated with 100,000 B16.F10 melanoma cells in 100 *μ*L phosphate-buffered saline under anesthesia by inhalation of isoflurane/O_2_. The mice were monitored daily for behavior and every other day for body weight. Mice were randomly divided into three experimental groups from which tumors were harvested at three different stages of tumor growth: 25 ± 0.7 (early stage/small volume, day 9 after tumor inoculation), 182 ± 1.5 (intermediate stage/volume, day 12–16), and 524 ± 42.3 (late stage/large volume, day 14–25) mm^3^. Tumors were measured every other day by calipers, and the volume was calculated according to the formula: tumor volume = 0.52 ∗ length ∗ width^2^, with “width” being the shorter of the two diameters. Animals were sacrificed under anesthesia by inhalation of isoflurane/O_2_, and tumors were excised, immediately snap frozen in liquid N_2_, and stored at −80°C until further analysis.

The animal experiments were approved by the local Animal Care and Use Committee of the University of Groningen.

### 2.2. Gene Expression Analysis by Quantitative RT-PCR

Extraction of total RNA from cryostat-cut sections of the tumors was carried out according to the protocol of RNeasy Mini Plus kit (Qiagen, Leusden, The Netherlands). RNA was analyzed qualitatively by gel electrophoresis and quantitatively by Nanodrop ND-100 spectrophotometry (NanoDrop Technologies, Rockland, DE, USA) and consistently found to be intact and protein-free. Total RNA was subsequently reverse transcribed as described previously [11], using Superscript III Reverse Transcriptase (Invitrogen, Carlsbad, CA, USA) in a 20 *μ*L final volume containing 250 ng of random hexamers (Promega, Madison, WI, USA) and 40 units of RNase OUT inhibitor (Invitrogen). mRNA expression analysis was performed in a real-time PCR-based, custom-designed, Low-Density Array set-up (Applied Biosystems, Foster City, CA, USA). The Low-Density Array was designed with exon-overlapping primers and minor groove-binding (MGB) probes of 46 genes (see Table  1 in supplementary material available online at doi:10.5402/2011/409308) selected for their involvement in angiogenesis, inflammation and basic influence on endothelial cell behavior, as reported in the literature [12–19], including GAPDH as a housekeeping gene. The Low-Density Array card was processed according to the suppliers' protocol and analyzed in an ABI PRISM 7900HT Sequence Detector (Applied Biosystems). For a selection of genes and for PCNA, the Notch family genes, *α*SMA and desmin (see Supplementary Table  1), real-time PCR was performed in duplicate per sample with 1 *μ*L cDNA per reaction in TaqMan PCR MasterMix in a total volume of 10 *μ*L, with primer-probe sets (where appropriate corresponding to the ones pre-spotted on the Low-Density Array cards) being purchased as Assay-on-Demand from Applied Biosystems (Nieuwekerk a/d IJssel, The Netherlands).

### 2.3. Laser Microdissection of B16 Tumor Vasculature

Five *μ*m cryosections mounted on polyethylene-naphtalene membranes attached to normal glass slides (P.A.L.M. Microlaser Technology AG, Bernried, Germany) were fixed in acetone and stained with Mayer's haematoxylin, washed with diethyl pyrocarbonate-treated water, and air-dried. Tumor vascular segments (1-2 × 10^6^ *μ*m^2^ surface area, including vessel lumen area) of both intermediate and large tumors and normal mouse kidney postcapillary venule segments (0.6 × 10^6^ *μ*m^2^) were microdissected using the Laser Robot Microbeam System (P.A.L.M. Microlaser Technology), yielding isolated vascular tissue from vessels with a clearly visible lumen. To isolate vascular segments from small profiles that do not contain a visible lumen and are therefore not identifiable based on haematoxylin staining, we applied fluorescence guidance during laser dissection. Tumor vascular segments representing small vascular profiles and large lumen-containing vessels were separately microdissected using an LMD6000 Laser Microdissection system (Leica, Wetzlar, Germany) from 9 *μ*m cryosections of large tumors mounted on polyethylene-terephthalate membranes on steel frames (Leica) that were stained with FITC-conjugated Griffonia Simplicifolia Lectin I IsolectinB4 (GSLI-isolectin B4: Vector Labs, Burlingame, CA, USA). The two vascular morphologies were distinguished based on the combination of size of the vessel (small versus large) and the absence respectively presence of a visible lumen.

Total RNA was extracted according to the protocol of Absolutely RNA Microprep kit (Stratagene, Amsterdam, The Netherlands) or RNeasy Micro Kit (Qiagen) for haematoxylin-stained lumen-containing vessels and fluorescent-stained vascular profiles/segments respectively, and reverse transcribed and analyzed by Low-Density Array and/or quantitative PCR as described above.

### 2.4. Immunohistochemical and Immunofluorescent Staining of Tumor Tissues

Immunohistochemical detection of antigens (CD31, ICAM-1, VCAM-1, Tie2, and vWF) in acetone-fixed, 5 *μ*m cryostat-cut sections was performed using the Dako Envision System-HRP kit (Dako Cytomation, Glostrup, Denmark) according to the suppliers' protocol. In brief, after rehydration, sections were incubated with endogenous peroxidase block diluted 1 : 1 with PBS for 5 minutes, washed with PBS and subsequently incubated with primary antibody diluted in 5% FCS in PBS for 60 minutes. The following primary antibodies were used: Rat-anti-Mouse CD31 IgG2a (BD Pharmingen; clone MEC13.3), Rat-anti-ICAM-1 IgG2b (YN1/1.7.4 hybridoma supernatant), Rat-anti-Mouse VCAM-1 IgG2a (BD Pharmingen; clone 429), Rat-anti-Tie2 (eBioscience; clone Tek4), and Rabbit-anti-vWF (Dako). In case of rat primary antibodies, sections were incubated with unlabeled Rabbit-anti-Rat IgG diluted 1 : 500 (H+L, Vector Labs) for 45 minutes. After incubation with anti-rabbit HRP-conjugated polymer for 30 minutes, peroxidase activity was detected with 3-amino-9-ethylcarbazole (AEC) complex and sections were counterstained with Mayer's haematoxylin (Merck, Darmstadt, Germany).

Expression of the nuclear proliferation marker Ki67 was assessed by immunohistochemical staining of acetone-fixed cryosections. Sections were incubated with rabbit monoclonal antibody to Ki67 (Lab Vision, Duiven, The Netherlands) in 5% FCS/PBS for 60 minutes, after Avidin/Biotin blocking (Dako). Sections were incubated with biotin-conjugated Goat-anti-Rabbit immunoglobulins (Dako) and subsequently incubated with Streptavidin-HRP (Dako) according to the manufacturers' protocol. Peroxidase activity was detected with AEC (Sigma-Aldrich; Steinheim, Germany) and sections were counterstained with Mayer's haematoxylin. Sections were embedded in Kaisers Glycerin (Merck) and examined using a Leica DMLB microscope and Leica Qwin V3 software.

The presence of pericytes was visualized by immunofluorescent double staining for *α*-Smooth Muscle Actin (*α*SMA) or desmin with CD31. Five *μ*m cryosections were fixed with acetone/methanol (1 : 1) on ice for 5 minutes and preincubated with PBS supplemented with 1% BSA, 10% normal goat serum (NGS; Dako), and 0.20% Tween-20 for 30 minutes at room temperature. Subsequently, sections were incubated with Cy3-conjugated mouse-anti-*α*SMA (Sigma) or Rabbit-anti-desmin (Abcam, Cambridge, UK) in the presence of Rat-anti-Mouse CD31 for 60 minutes at room temperature. All antibody incubations were performed in PBS supplemented with 1% BSA, 10% NGS, and 0.20% Tween-20. Sections were washed with PBS and subsequently incubated with Alexa fluor 568-conjugated Goat-anti-Rabbit IgG to detect desmin and Alexa fluor 488-conjugated Goat-anti-Rat IgG to detect CD31 (both H+L, Molecular Probes Invitrogen Detection Technologies, Eugene, Oregon). After washing with PBS, nuclear counterstaining was performed using DAPI (F. Hoffmann-La Roche Ltd, Basel, Switzerland). Autofluorescence was reduced by incubating the sections in 0.1% Sudan Black (Sigma-Aldrich) in 70% ethanol for 30 minutes at room temperature. Sections were embedded in Citifluor (Citifluor Ltd., London, UK) and examined using a fluorescence microscope (DM RXA, Leica) and Leica Qwin V3 software.

### 2.5. Statistics

Linear Mixed Effects (LMEs) Models and Analysis to account for multiple measurements within mice were used for data analysis to address the significance of observed effects [20]. Multiple testing was controlled by the step-up False Discovery Rate-controlling procedure of Benjamini and Hochberg [21] in calculating adjusted *P* values (*P*_adj_ < 0.05 indicated statistical significance) for the different research questions. All analyses were performed using *R* [22].

Statistically significant differences in gene expression in endothelium of the two different vascular morphologies observed were assessed by means of Student's *t*-test (GraphPad Prism, GraphPad Software, San Diego, CA) and considered to be significant when *P* < 0.05.

## 3. Results

### 3.1. Different B16.F10 Growth Stages Presented with a Different Vascular Morphology, but with a Similar Gene Expression Profile

**Figure 1:**
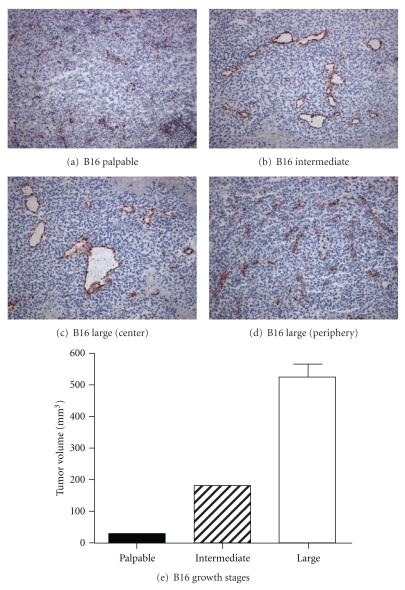
Morphological appearance of tumor vasculature of B16.F10 melanoma growing subcutaneously: immunohistochemical detection of CD31. Tumors were harvested at three different growth stages, based on tumor volumes as depicted in (e) (mean of 3 mice per group ± SEM). Vascular morphology differed per tumor growth stage, presenting vessels with increasing lumen size in intermediate and large tumors next to the presence of small vascular profiles, while small tumors were characterized by only small vessels that were generally lacking a visible lumen (Magnification 100x).

We hypothesized that early-stage tumor growth would be characterized predominantly by angiogenic sprouting vasculature, while in late-stage tumors active angiogenic vascular sprouts would exist next to “quiescent” matured neovessels. This would represent one mode of vascular heterogeneity that may affect efficacy of drug treatment. Therefore, we first investigated neovessel development during tumor growth from a morphological point of view and at a molecular level in s.c. growing B16 tumors, which were harvested at an early (palpable), intermediate, and late stage of tumor development (Figure 1).

**Figure 2:**
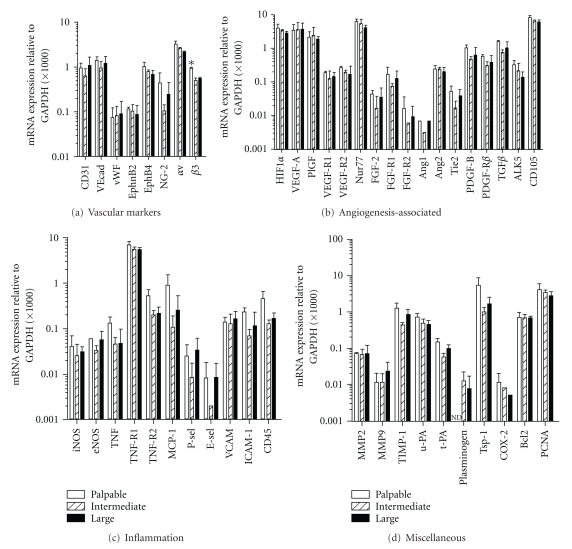
In the three different stages of s.c. B16.F10 tumor growth, mRNA expression of the 46 genes under study is similar. Tumors were harvested at palpable, intermediate, and large volumes (early, intermediate and late stages). Values represent mRNA expression levels measured by qRT-PCR-based Low-Density Array and adjusted to GAPDH. Mean + SD; *n* = 3. Expression of FGF-2, FGFR2, Ang1, P-selectin, E-selectin, and COX-2 was additionally measured twice by manual qRT-PCR. PCNA was measured by manual real-time RT-PCR alone in duplicate. *P < 0.05. ND: not detectable.

Vascular morphology changed drastically throughout tumor development. While palpable tumors were characterized by small vessels, generally containing no or a small lumen, the vascular network of intermediate and large tumors consisted of vessels with wide lumen that existed next to small vascular structures without lumen. The vessels with the largest lumen surface were found in the large tumors (Figures 1(a)–1(d)). Strikingly, these morphological differences were not accompanied by a major difference in angio-gene expression profile (Figure 2), except for the expression of integrin *β*3, which was statistically significantly higher in small tumors versus intermediate and large tumors (*P* < 0.05). Expression of molecules from the VEGF-family and their receptors, regarded as key regulators of the first step of the angiogenic cascade, was equal in the three stages of tumor outgrowth. The ratio of Ang2/Ang1 mRNA levels varied between 24 and 78, which is in line with the current dogma that a dysbalance in Ang2/Ang1 in favor of Ang2 de-stabilizes the endothelium and presensitizes it for (VEGF-induced) proliferation and angiogenic sprouting [23].

**Figure 3:**
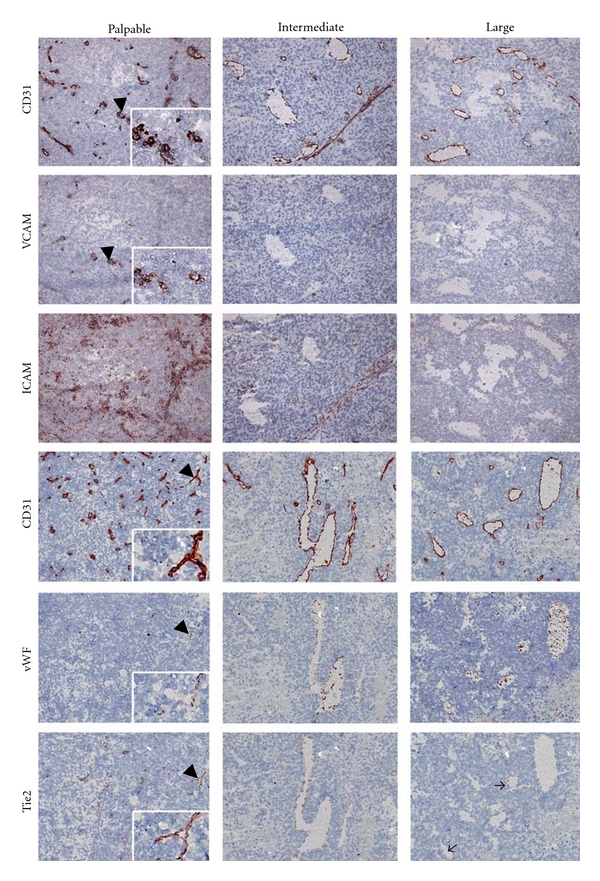
Vascular localization and expression of endothelial molecules at the protein level in B16.F10 tumors growing at three different stages of tumor growth. Expression of VCAM-1 and ICAM-1 was highest in the early-stage tumors, as compared to intermediate and large tumors. A granular pattern of vWF expression was found in a small number of tumor vessels; the majority of them were negative for vWF. Tie2 expression remained the same throughout tumor development and was observed in only a subset of the vessels. The top three and bottom three rows of panels represent immunohistochemical staining of consecutive sections. Arrowheads point to the structures that are enlarged in the insets. Thin arrows point to vessels in the large tumor that are positive for Tie2, while the largest vessel in the same photograph did not express detectable levels of Tie2 protein. Pictures are representative examples of each experimental group (Magnification 100x).

Low mRNA levels of the adhesion molecules P- and E-selectin were associated with nondetectable protein levels (data not shown), while the mRNA expression levels of VCAM-1 and ICAM-1 were higher and associated with protein expression restricted to a subset of blood vessels (Figure 3). ICAM-1 was furthermore expressed by perivascular infiltrates of some vessels. Both VCAM-1 and ICAM-1 expression were more intense in palpable compared to intermediate and large tumors, a trend also observed at the ICAM-1 mRNA level that was however statistically not significant. Interestingly, the mRNA level of von Willebrand Factor, which is like CD31 and VE-cadherin regarded as an endothelial marker molecule and often used as research tool for immunofluorescence double staining protocols [24], was much lower than that of CD31, an observation that was confirmed at the protein level. In contrast to CD31, vWF protein expression appeared granular and covered only parts of the vessel wall of a small subset of vessels. Also Tie2 expression was detected in a subset of vessels and often did not fully cover the endothelial lining within the vessel wall. Both vWF and Tie2 protein expression patterns did not change during tumor growth.

### 3.2. Gene Expression in the B16.F10 Tumor Vascular Compartment That Represents Lumen-Containing Vessels

We hypothesized that vascular behavior is the result of locally controlled balances of gene expression. As the majority of the genes under study are not restricted to the endothelium, we zoomed in on the local balances of vascular gene expression by isolating tumor vascular segments from their environment by laser microdissection prior to gene expression analysis.

**Figure 4:**
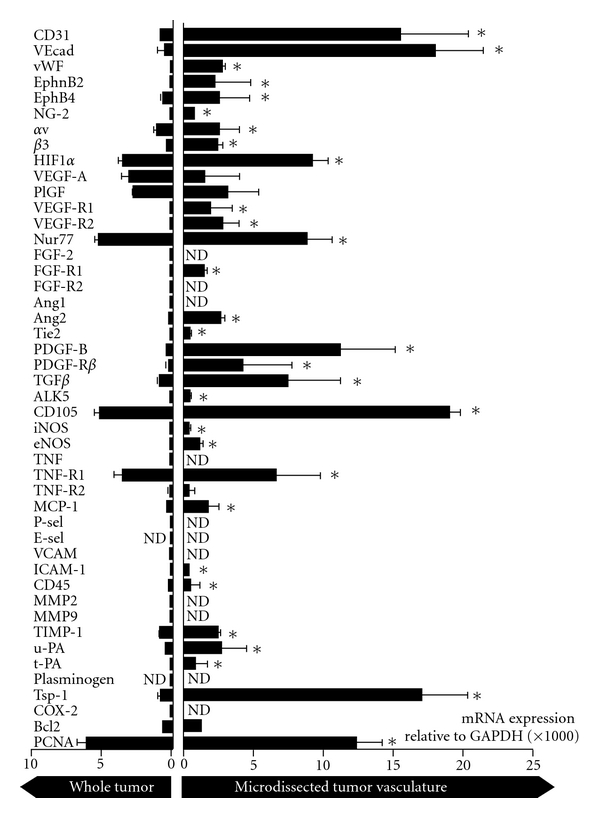
Vascular compartmentalization of angio-gene expression in a large tumor. Tumor vasculature was laser microdissected and its gene expression profile was compared to that of the tumor as a whole. Values represent mRNA levels adjusted to GAPDH. Mean values ± SD of 3 qPCR analyses, except for CD31, VE-cadherin, vWF, EphrinB2, EphB4, Integrin *α*v, VEGFR2, Tie2, iNOS, eNOS, TNF*α*, TNFR1, TNFR2 (a total of 5 qPCR analyses), and for VEGF (a total of 7 qPCR analyses) and PCNA (qPCR in quadruplicate). *P < 0.05. ND: not detectable.

mRNA of microdissected tumor vasculature identified by light microscopy in haematoxylin-stained biopsies, hence representing lumen-containing vasculature, showed a ~20–60 fold enrichment in endothelial marker genes CD31, VE-cadherin, vWF, and EphrinB2, compared to mRNA isolated from a tumor biopsy as a whole (Figure 4). Moreover, we could assign VEGFR1 and -R2, FGFR1, Ang2, Tie2, PDGF-B, PDGF-R*β*, eNOS, t-PA, and Tsp-1 as being tumor vascular-associated in this s.c. B16.F10 tumor model, as they exhibited a similar 15–50-fold enrichment. In contrast, FGF-2, FGFR2, Ang1, MMP2, MMP9, plasminogen, TNF*α*, P-selectin, E-selectin, and COX-2 transcripts were not detectable in this compartment.

Of note is the observation that compartmentalization of genes revealed that the relative balances of gene expression in the tumor vasculature differed from those observed when analyzing the tumor tissue as a whole. For example, when analyzing the mRNA levels in the tumors, the levels of integrin *β*3 were 2.5-fold lower than those of integrin *α*v, while in the tumor vasculature both integrin subunits were expressed at equal levels. Similarly, the ratio between the pro-apoptotic gene Tsp-1 [25] and the antiapoptotic Bcl2 shifted from 1.9 in the tumor to 13.6 in the tumor vasculature, which may point to a relatively higher degree of Tsp1-induced proapoptotic signaling in the tumor vasculature compared to the non-vascular tumor compartment.

### 3.3. Different Vascular Morphologies Are Associated with a Similar Profile of Angiogenic Gene Expression

**Figure 5:**
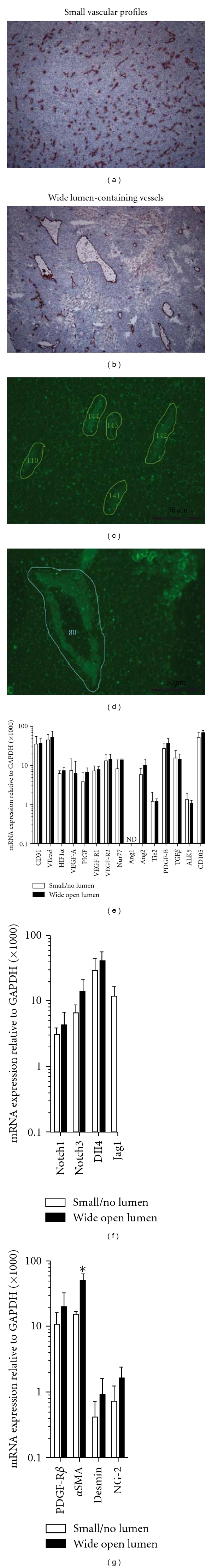
Different vascular morphologies displayed a similar pattern of gene expression. High variability of vascular morphology was observed in tumors of large volume. Large lumen-containing vessels ((b, d) vessel lumen size > 25 *μ*m) existed next to much smaller blood vessels that did not contain a visible lumen (a, c). (a, b) Immunohistochemical staining for CD31: pictures represent different regions within the same large tumor (Magnification 100x). (c, d) Cryosections from large tumors were stained with GSL-I IsolectinB4:FITC and the different vascular morphologies were separately isolated by laser microdissection along the depicted lines, magnification 400x. Microdissection of these different vascular phenotypes followed by gene expression profiling did not reveal a differential pattern of mRNA expression of angiogenesis- and vascular stability-regulating genes (e), nor of the Notch family of genes that has recently been identified to be important regulators of both angiogenic sprouting and endothelial-pericyte adhesion (f), but demonstrated that the lumen-containing vessels were associated with significantly higher levels of *α*SMA mRNA compared to the small vessels without lumen (g). (e)–(g) Mean values + SD of duplicate qPCR measurements of three large tumors. ND: not detectable. *P < 0.05.

The vasculature of late-stage tumors was characterized by regional variations in vascular morphology. Regions with small vascular profiles present at a high density (Figure 5(a)) existed next to regions with large vessels containing a wide lumen that were distributed at a much lower density (Figure 5(b)). Based on reports describing a role for HIF-1*α* and VEGF in determining blood vessel morphogenesis, including lumen formation [26, 27], we hypothesized that a differential pattern of expression of these genes might underlie the different vascular morphologies in this tumor model. To study this, we separately microdissected large, lumen-containing vessels and small vascular segments without lumen, guided by fluorescence staining with the FITC-labeled GSLI-isolectinB4 (Figures 5(c) and 5(d)). Surprisingly, both vascular morphologies were characterized by an identical profile of mRNA expression of those genes that were previously assigned as major controllers of vascular morphology and structure (Figure 5(e)). Also mRNA levels of members of the Notch family of genes and their ligands, which were recently identified as being in control of angiogenic sprout formation (Notch1 and Dll4 [28]) on the one hand, and endothelial-pericyte adhesion (Notch3 and Jag1 [29]) on the other hand, did not differ in the two morphologically different vascular segments (Figure 5(f)).

### 3.4. Different Vascular Morphologies Were Associated with Different Pericyte Phenotypes

**Figure 6:**
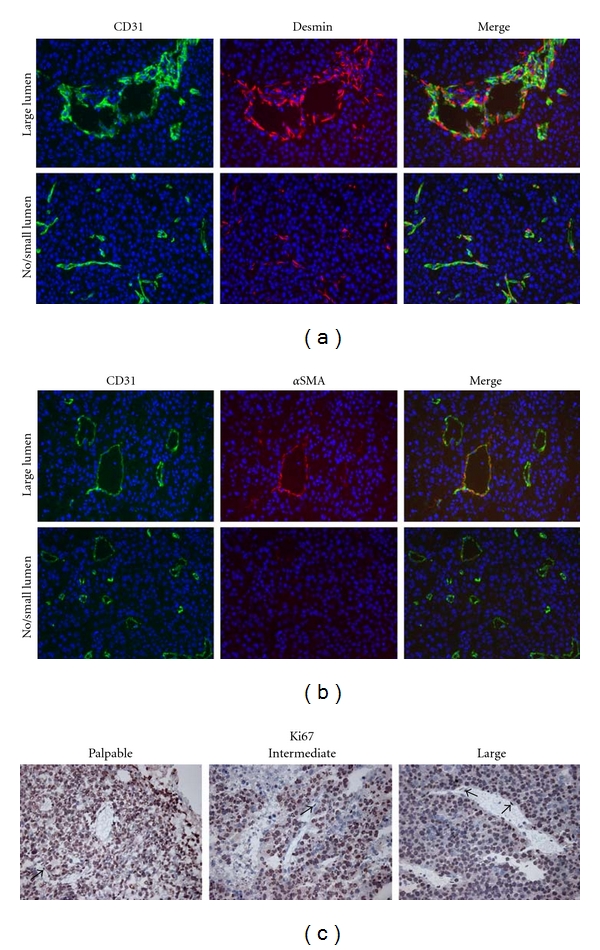
Vessels with different morphological appearance are covered with pericytes presenting with a different pattern of pericyte marker expression. (a, b) Immunofluorescent double staining for CD31 (green) and desmin (red) or *α*-SMA (red) and nuclear counter stain (DAPI; blue) of a representative tumor from the group of late-stage, large volume tumors (Magnification 200x). (c) Immunohistochemical detection of Ki67 revealed homogeneous distribution of proliferative activity throughout the whole tumor, in all three stages of tumor growth (Magnification 200x). Arrows point to Ki67-positive endothelial nuclei.

As pericyte coverage can affect vascular morphology, we next set out to investigate pericyte marker expression in these two segments. Of the three markers studied (*α*SMA, desmin, and NG-2), mRNA levels of *α*SMA were found to be more than 3-fold higher in large lumen-containing vessels compared to small vascular profiles, while the other two genes did not significantly differ (Figure 5(g)). To validate this observation we determined the extent and pattern of pericyte coverage of these two vascular profiles by immunofluorescent double staining for CD31 and either *α*SMA or desmin as pericyte markers. This demonstrated that lumen-containing B16 tumor vessels were associated with pericytes that stained positive for both desmin and *α*SMA, while the majority of small vessels were associated with support cells that expressed only desmin. These protein data corroborated the difference in mRNA expression in the two morphologically different vascular beds within the same tumor (Figure 6).

Absence of *α*SMA was previously shown to be a feature of a microvasculature that is actively engaging in angiogenesis [30, 31]. This implies that *α*SMA-lacking vessels are most likely to be found in areas where angiogenesis is a prerequisite for growth, *that is,* in areas where tumor cells are highly proliferative. To address whether such a process indeed underlies the observed differences, we assessed the proliferative activity of endothelial cells of both types of vascular morphologies, and of their surrounding tumor cells. While the majority of tumor cells were proliferative active, Ki67 expression was only sporadically detected in endothelial cells of both the small vascular profiles and the lumen-containing vessels. Ki67 expression by tumor cells, furthermore, appeared homogeneously distributed throughout the tumor, irrespective of the stage of tumor growth, and could not be related to the observed differences in vascular morphology nor vascular maturity. Similarly, PCNA mRNA levels did not differ in small vascular profiles compared to lumen-containing large vessels (data not shown).

### 3.5. The Gene Expression Profiles of the Tumor Vasculatures Represent an Active, Angiogenic Phenotype: Comparison with Quiescent Endothelium

**Figure 7:**
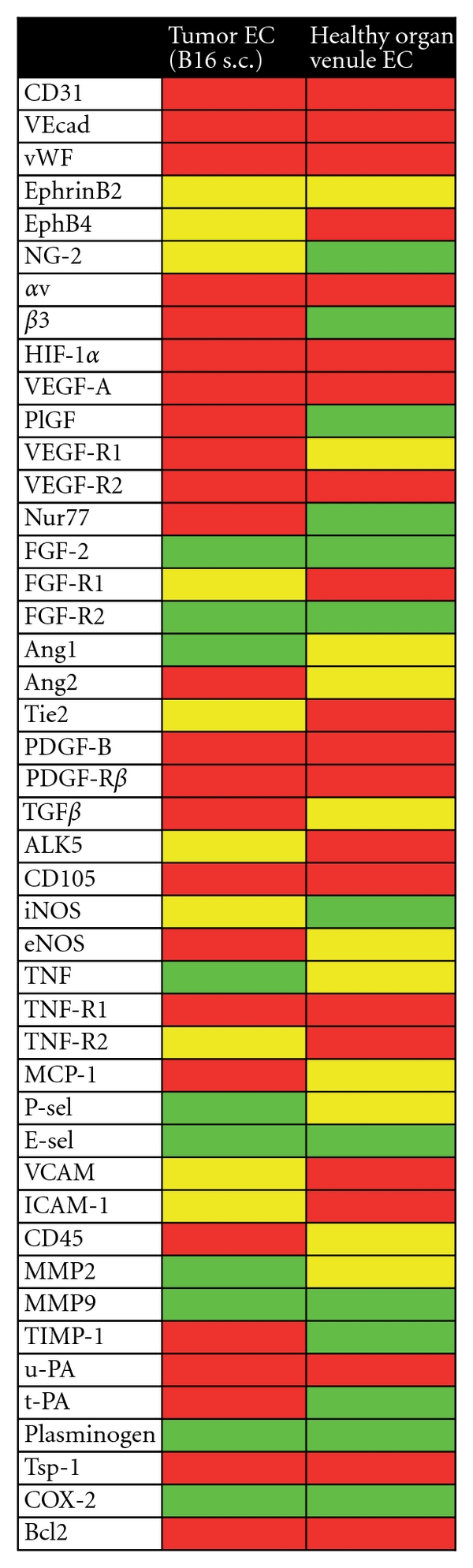
Summary of gain and loss of gene expression in the B16.F10 tumor vascular compartment of intermediate and large tumors compared to normal quiescent endothelium: gene expression in microdissected tumor endothelium from s.c. growing intermediate and large B16.F10 tumors (a total of 6 animals) and microdissected kidney venule endothelium from healthy C57bl/6 mice (10 animals). Red: detectable expression in more than 75% of all analyzed samples; yellow: detectable expression in 25–75% of all analyzed samples; green: expression of the gene could not be detected in the majority of the samples (less than 25% of positivity).

To molecularly describe the difference in phenotype between B16.F10 tumor vasculature that actively engages in angiogenesis, and quiescent, non-angiogenic vasculature, we profiled the expression of the angio-genes in normal postcapillary venule endothelium microdissected from a healthy mouse kidney (Figure 7). This analysis revealed that integrin *β*3, PlGF, Nur77, TIMP-1, and t-PA were absent in the majority of normal healthy kidney endothelium samples, while being present in the tumor endothelium. Likewise, VEGFR1, Ang2, TGF*β*, eNOS, and MCP-1 were more frequently detected in the tumor endothelium as compared to normal kidney endothelium. On the opposite, EphB4, FGFR1, Tie2, Alk5, TNFR2, and the adhesion molecules VCAM-1 and ICAM-1 were more frequently detected and at higher levels in normal kidney endothelium as compared to tumor endothelium from intermediate and large tumors.

## 4. Discussion

In this study we investigated the angiogenic make-up of s.c. growing B16.F10 tumors at different stages of tumor growth. We furthermore mapped the molecular angiogenic make-up of the tumor vascular compartment and related that to the morphology of the vasculature. Our focus lay on genes that were previously shown to be responsible for the induction of angiogenic sprouting and vascular maturation. We showed that, although the vascular morphology changed dramatically during tumor outgrowth, this was not associated with major differences in expression of the 46 genes analyzed. Moreover, morphologically highly different tumor vessels exhibited similar expression profiles of the major angio-genes studied. The expression of *α*SMA was a discriminative parameter for large vessels; its high mRNA levels and clear protein expression in large lumen-containing vessels contrasted with the much lower mRNA levels and the absence of protein in small vascular profiles without a lumen. This difference in vascular support cell/pericyte characteristics may underlie the difference in vascular morphology in this tumor model; yet the angio-genes accompanying this phenotype remain until now unknown. We demonstrated that the 46 genes chosen are associated with the angiogenic process by comparing their expression in the tumor vascular compartment with that in a mature vessel of a healthy organ, which showed that many proangiogenic genes indeed exhibited a gain of function in the tumor vasculature. From this study we conclude that the vasculature of s.c. B16.F10 tumors continues to actively engage in angiogenesis throughout the different stages of tumor growth. The striking differences in vascular morphology between small and large tumors are associated with a difference in pericyte activation but have no direct relation to the transcriptional activity of the angio-genes analyzed in this study. A similar pattern of proangiogenic gene expression continues to exist in even later B16.F10 growth stages (tumor volumes ranging from 461–1409 mm^3^; data not shown).

Our aim was to investigate how in a growing tumor of a widely employed mouse model the expression of genes important in the control of the different stages of angiogenesis and vascular maturation would spatiotemporally change with ongoing tumor growth. Surprisingly, our findings demonstrated that the B16.F10 tumor does not change its molecular angiogenic repertoire during tumor development. This outcome contrasts findings in a rat C6 glioma model growing in the brain [8] as well as in murine orthotopic KM12SM colon carcinoma growth [9] in which VEGF expression varied during tumor growth progression. Furthermore, expression of Ang2 has been shown to be elevated during the early stages of tumor outgrowth in a U-87 MG glioma model growing intracranially in athymic mice, while being downmodulated once the vascular structures of the tumor had established, as in the later stages of tumor outgrowth [32]. The here described elevated levels of the VEGF-receptors, Nur77, Ang2, and Tie2 in the tumor vascular compartment compared to the tumor compartment [16, 23], as well as the significantly higher level of PDGF-B, a molecule extensively produced by angiogenic tip cells on a sprouting tip [33], suggest a VEGF/Ang2-driven proangiogenic phenotype of the B16.F10 model employed. Moreover, as compared to normal endothelium, tumor endothelium showed gain of function of numerous proangiogenic molecules such as integrin *β*3, PlGF, VEGFR1, Nur77, Ang2, eNOS, TGF*β*, MCP-1, TIMP-1, and t-PA, while loss of function was observed for vessel stability genes such as Ang1 and Tie2. From this, it can be concluded that throughout the growth period studied, the majority of vessels of B16.F10 tumors remain engaged in the angiogenic process. Interestingly, the same pattern of proangiogenic gene expression was found when B16.F10 tumors were grown in the highly vascularized brain (Supplementary Figure  1), demonstrating that neither the host environment where the tumor is growing changes the proangiogenic molecular repertoire of the tumor vasculature.

Yet, during s.c. B16.F10 outgrowth, the vascular morphology underwent a major shift from small, lumen-less vessels mainly covered by desmin-positive pericytes to large lumen-containing vessels mainly covered by pericytes positive for both *α*SMA and desmin. Recently, Helfrich et al. reported that spontaneously arising MT/ret transgenic melanomas were characterized by a similar distribution of *α*SMA- and desmin-positive vessels in relation to vascular lumen size and density and found that *α*SMA-covered large vessels were less sensitive to anti-VEGF therapy [31], thereby illustrating the consequences of tumor vascular heterogeneity for the outcome of antiangiogenic therapy. A similar heterogeneity in vascular morphology as observed in our study was previously reported by Kashiwagi et al. in B16.F10 tumors grown in cranial windows. In their study, changes in vascular morphology could not be related to transcriptional regulation of 96 angiogenesis-associated genes, including the Angiopoietins and Tie2, VEGF and its receptor, and PDGF [34]. Vascular morphology has previously been shown to be under the control of these genes, with low VEGF levels resulting in a reduction of blood vessel lumen size in human gastric TMK-1 tumors in athymic nude mice [26] and in the chick CAM assay [27]. In the B16.F10 tumor model in immune-competent mice employed here, vascular morphology did however not relate to HIF-1*α* and VEGF mRNA levels. The expression levels of the VEGFR2 response gene Nur77 in the three growth stages as well as in the two different tumor vessel morphologies furthermore corroborate the conclusion that similar levels of VEGF-activity can exist while vasculatures are morphologically highly variable. Another set of genes previously shown to affect vascular morphology includes Notch and Notch ligand Dll4 [28, 35]. Enhanced Dll4 expression in xenograft models was associated with increased vessel size [36], but in our model, these genes were not discriminative between small vascular profiles and lumen-containing vessels. From these data it becomes clear that each preclinical angiogenesis model needs to be appreciated for its own molecular repertoire underlying angiogenesis and vascular remodeling and that care should be taken in extrapolating observations from one model to the other.

Although we did not achieve the broader aim of the study, that is, identification of the molecular nature of tumor vascular heterogeneity during tumor outgrowth, a number of issues were revealed that are of relevance when studying angiogenesis. One important finding is that vWF expression showed a remarkable heterogeneity throughout the tumor vasculature. It was not expressed in a pan-endothelial manner as were CD31 and VE-cadherin. A similar loss of vWF expression during angiogenesis was reported previously by us in a mouse model of inflammation [37], as well as by Yano et al. in biopsies from Non-Small Cell Lung Cancer patients [38]. The use of vWF as a marker for tumor endothelium, as is employed in, for example, fluorescent double-labeling strategies [24], hence can have important drawbacks. Moreover, as endothelial cells are numerically underrepresented in a tumor, their molecular profile will be masked in whole tumor mRNA isolates by that of the dominating tumor cells. By applying laser microdissection-assisted isolation of the tumor vasculature prior to transcriptional profiling, we were able to reveal local balances in gene expression that are controlled by the pathophysiological environment which would have remained masked when analyzing whole tumor RNA samples. Although microdissection of tumor endothelium does not yield a pure endothelial isolate as visualized by detection of mRNA impurities such as pericyte-associated genes NG-2, *α*SMA, and desmin, judging from the small enrichment of these molecules relative to that of CD31 and VE-cadherin, their contribution to the population of dissected cells is minor.

Our results indicate that B16.F10 melanoma vascular development is accompanied by major changes in the vascular morphology. This observation has clinical relevance, as variations in vascular architecture have been described in biopsies from prostate carcinoma patients with metastasis in bone, liver, and lymph node [39]. Human clear-cell renal cell carcinoma is characterized by heterogeneous tumor vascular behavior, including variations in microvascular density, endothelial cell proliferation, and expression of angiogenic growth factors [40]. Together, these examples emphasize the clinical relevance of the occurrence of heterogeneity of tumor vascularization and the need for in-depth studies on the molecular nature thereof. Ideally, the technique of sampling tumor vascular segments from the pathophysiological environment would allow to microdissect immunohistochemically identified subsets of pericytes or endothelial cells that are differentially activated as observed by, for example, gain and loss of VEGFR2 and *α*v*β*3. If such a technique could be combined with genome-wide transcriptional profiling, unraveling the true molecular nature of morphological changes and tumor vascular heterogeneity would come within reach. Immunofluorescence staining with the lectin as employed here is a first step, as it provides for the first time the opportunity to dissect endothelial cells that are difficult to visualize, while so far in our hands antibody-based protocols did not allow for proper visualization with maintenance of RNA integrity. Combination of laser microdissection with kinome and protein arrays to detect phosphorylation status of many kinases involved in angiogenic signaling, for example, VEGFR2, ERK, and Akt, would furthermore broaden the options to get a clearer view on the molecular basis of tumor vascular heterogeneity [41]; yet at present this is unfeasible due to the limited amount of tissue yielded by laser microdissection.

Future studies should focus on an extension of the mRNA expression profiles in the tumor vascular microenvironment, on regulation and (posttranslational) modification thereof at the protein level, and on determination of the consequence of antiangiogenic drug treatment on these parameters. Integrating this knowledge will ultimately contribute to the establishment of effective antiangiogenic therapy in the clinic and relevant biomarkers of efficacy.

## Supplementary Material

Supplementary Figure 1: B16.F10 vascular gene expression is similar in different host environment. To investigate the influence of the host environment on the tumor vascular gene expression, we compared gene expression of B16.F10 tumors growing in the avascular subcutaneous space with that of B16.F10 tumors growing in the highly vascularized brain. Therefore, 300,000 B16.F10 cells in 3 **μ**l PBS were stereotactically implanted in the left striate nucleus of male C57bl/6 mice, after anesthetizing with Ketamin/Medetomidin i.p. (75 mg/kg and 1 mg/kg respectively). Inoculation was performed at 2.5 mm lateral from the bregma and at 2.5 mm depth from the cortical surface, using a 10 **μ**l SGE syringe. At day 9 after tumor inoculation, the mice were sacrificed and brains (with tumor) were taken out and frozen. To separate the tumor tissue from the surrounding brain, we microdissected tumor tissue from tumor-containing brain sections using the Laser Robot Microbeam System (P.A.L.M. Microlaser Technology), yielding approximately 10x106 **μ**m^2^ tissue. Total RNA was extracted using Qiagen RNeasy Micro kit (Qiagen), reverse transcribed and analyzed by Low Density Array and/or quantitative PCR as described above. Comparing the gene expression profiles in subcutaneously and intracranially growing B16.F10 tumors (both harvested at day 9 after implantation) revealed a similar pattern. This demonstrated that the tumor host environment did not affect the proangiogenic molecular repertoire of the tumor vasculature.
Supplementary Table 1: List of genes assays by real-time RT-PCR. List of genes that were assayed by custom-designed Low-Density Array or manual qRT-PCR. Numbers refer to primer-probe sets spotted on the Low-Density array card or purchased as Assay-on-Demand from Applied Biosystems.

## Abbreviations

Ang1/2: Angiopoietin-1/2
Alk5: Activin receptor-like kinase-5
Bcl2: B-cell lymphoma protein-2
COX-2: Cyclooxygenase-2
eNOS: endothelial Nitrogen Oxide Synthase
FGF(-R1/2): Fibroblast Growth Factor (Receptor 1/2)
HIF-1*α*: Hypoxia inducible factor-1*α*
ICAM-1: Intercellular adhesion molecule-1
iNOS: inducible Nitrogen Oxide Synthase
MCP-1: Monocyte chemoattractant protein-1
MMP2/9: Matrix metalloproteinases 2/9
NG-2: Neuron glial-2
PCNA: Proliferating cell nuclear antigen
PDGF(-R*β*): Platelet-Derived Growth Factor (Receptor-*β*)
PlGF: Placental Growth Factor
*α*SMA: *α*-Smooth Muscle Actin
TGF*β*: Transforming Growth Factor-*β*
TIMP-1: Tissue inhibitor of metalloproteinase-1
TNF(-R1/2): Tumor Necrosis Factor (Receptor 1/2)
t-PA: tissue-type Plasminogen Activator
Tsp-1: Thrombospondin-1
u-PA: urokinase-type Plasminogen Activator
VCAM-1: Vascular cell adhesion molecule-1
VEGF(-R1/2): Vascular Endothelial Growth Factor (Receptor 1/2)
vWF: von Willebrand Factor.
