# Supplementary figures and images for: Tumor Vascular Morphology Undergoes Dramatic Changes during Outgrowth of B16 Melanoma While Proangiogenic Gene Expression Remains Unchanged
Source: ISRN Oncol. 2011 Dec 4;2011:409308. doi: 10.5402/2011/409308 (PMC3249352; doi:10.5402/2011/409308)

**A: Vascular markers**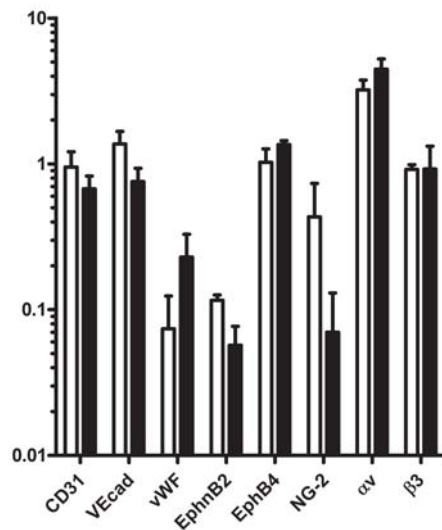**B: Angiogenesis-associated**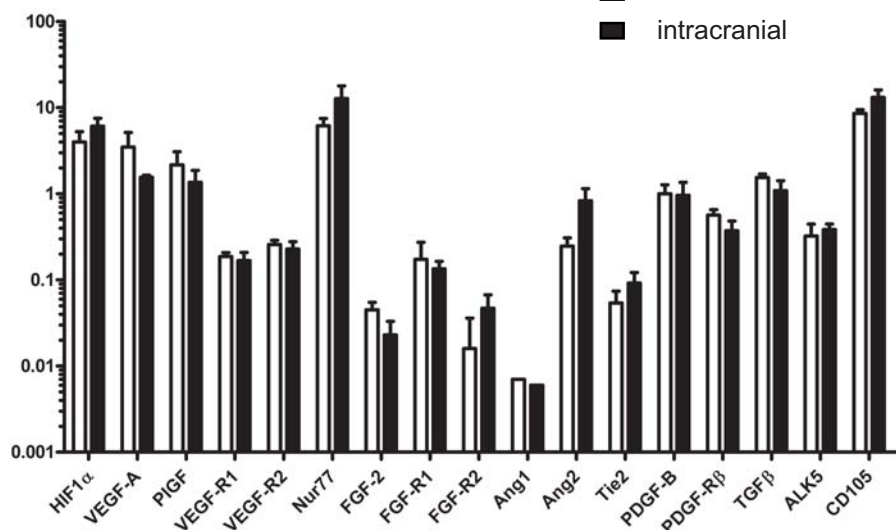**C: Inflammation**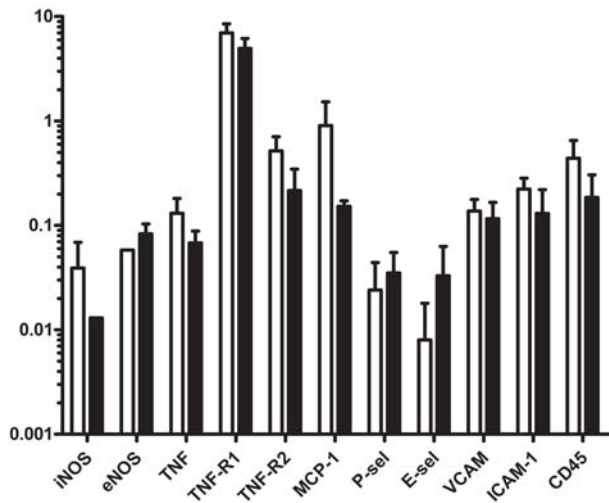**D: Miscellaneous**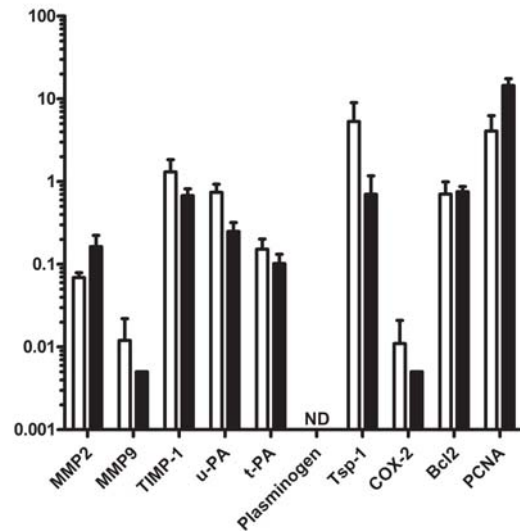

Supplement: Supplementary file 1 — Supplementary Figure 1: B16.F10 vascular gene expression is similar in different host environment. To investigate the influence of the host environment on the tumor vascular gene expression, we compared gene expression of B16.F10 tumors growing in the avascular subcutaneous space with that of B16.F10 tumors growing in the highly vascularized brain. Therefore, 300,000 B16.F10 cells in 3 μl PBS were stereotactically implanted in the left striate nucleus of male C57bl/6 mice, after anesthetizing with Ketamin/Medetomidin i.p. (75 mg/kg and 1 mg/kg respectively). Inoculation was performed at 2.5 mm lateral from the bregma and at 2.5 mm depth from the cortical surface, using a 10 μl SGE syringe. At day 9 after tumor inoculation, the mice were sacrificed and brains (with tumor) were taken out and frozen. To separate the tumor tissue from the surrounding brain, we microdissected tumor tissue from tumor-containing brain sections using the Laser Robot Microbeam System (P.A.L.M. Microlaser Technology), yielding approximately 10x106 μm2 tissue. Total RNA was extracted using Qiagen RNeasy Micro kit (Qiagen), reverse transcribed and analyzed by Low Density Array and/or quantitative PCR as described above. Comparing the gene expression profiles in subcutaneously and intracranially growing B16.F10 tumors (both harvested at day 9 after implantation) revealed a similar pattern. This demonstrated that the tumor host environment did not affect the proangiogenic molecular repertoire of the tumor vasculature. Supplementary Table 1: List of genes assays by real-time RT-PCR. List of genes that were assayed by custom-designed Low-Density Array or manual qRT-PCR. Numbers refer to primer-probe sets spotted on the Low-Density array card or purchased as Assay-on-Demand from Applied Biosystems. [file 409308.f1.pdf]
