# Supplementary material for: Tumor Vascular Morphology Undergoes Dramatic Changes during Outgrowth of B16 Melanoma While Proangiogenic Gene Expression Remains Unchanged
Source: ISRN Oncol. 2011 Dec 4;2011:409308. doi: 10.5402/2011/409308 (PMC3249352; doi:10.5402/2011/409308)
Supplement: Supplementary file 2 [file 409308.f2.pdf]

Supplementary Table 1: List of genes assayed by real-time RT-PCR.

Functional category	***Gene***	***Assay number***
Vascular markers		
Endothelial cell marker genes	CD31	Mm00476702_m1
	VEcad	Mm00486938_m1
	vWF	Mm00550376_m1
	EphnB2	Mm00438670_m1
	EphB4	Mm00438750_m1
Pericyte marker gene	NG-2	Mm00490179-m1
	αSMA	Mm01546133_m1
	Desmin	Mm00802455_m1
Integrins	αv	Mm00434506_m1
	β3	Mm00443980_m1
Angiogenesis		
Angiogenesis, vessel de-stabilization	HIF-1α	Mm00468869_m1
	VEGF-A	Mm00437304_m1
	PlGF	Mm00435613_m1
	VEGFR1	Mm00438980_m1
	VEGFR2	Mm00440099_m1
	Nur77	Mm00439358_m1
	FGF-2	Mm00433287_m1
	FGFR1	Mm00438923_m1
	FGFR2	Mm00438941_m1
Vessel stability	Ang1	Mm00456503_m1
	Ang2	Mm00545822_m1
	Tie2	Mm00443242_m1
	PDGF-B	Mm00440678_m1
	PDGFRβ	Mm00435546_m1
	TGFβ	Mm00441724_m1
	ALK5	Mm00436964_m1
	CD105	Mm00468256_m1
Vascular morphogenesis/Notch family	Notch1	Mm00435245_m1
	Notch3	Mm01345646_m1
	Dll4	Mm00444619_m1
	Jag1	Mm00496902_m1
Inflammation		
NOS	iNOS	Mm00440485_m1
	eNOS	Mm00435204_m1
Inflammatory cytokines	TNF	Mm00443258_m1
	TNFR1	Mm00441875_m1
	TNFR2	Mm00441889_m1
	MCP-1	Mm00441242_m1
Adhesion molecules	P-sel	Mm00441295_m1
	E-sel	Mm00441278_m1
	VCAM	Mm00449197_m1
	ICAM-1	Mm00516023_m1
Leukocytes	CD45	Mm00448463_m1
Miscellaneous		
ECM degradation	MMP2	Mm00439508_m1
	MMP9	Mm00442991_m1
	TIMP-1	Mm00441818_m1
	u-PA	Mm00447054_m1
	t-PA	Mm00476931_m1
	Plasminogen	Mm00447087_m1
Endogenous anti-angiogenic	Tsp-1	Mm00499022_m1
Survival	Bcl2	Mm00477631_m1
Prostaglandin synthesis	COX-2	Mm00478374_m1
Proliferation	PCNA	Mm00448100_g1
House-keeping gene	GAPDH	Mm99999915_g1
